# Region-Specific Changes of Insular Cortical Thickness in Heavy Smokers

**DOI:** 10.3389/fnhum.2019.00265

**Published:** 2019-07-31

**Authors:** Fuchun Lin, Guangyao Wu, Ling Zhu, Hao Lei

**Affiliations:** ^1^National Center for Magnetic Resonance in Wuhan, State Key Laboratory of Magnetic Resonance and Atomic and Molecular Physics, Wuhan Institute of Physics and Mathematics, Chinese Academy of Sciences, Wuhan, China; ^2^University of Chinese Academy of Sciences, Beijing, China; ^3^Department of Radiology, Zhongnan Hospital, Wuhan University, Wuhan, China; ^4^Department of Medical Imaging, Shenzhen University General Hospital, Medical College of Shenzhen University, Shenzhen, China

**Keywords:** insula, cortical thickness, heavy smokers, region-specific changes, structural MRI

## Abstract

Insula plays an essential role in maintaining the addiction to cigarette smoking and smoking-related alterations on the insular volume and density have been reported in smokers. However, less is known about the effects of chronic cigarette smoking on the insular cortical thickness. In this study, we explored the region-specific changes of insular cortical thickness in heavy smokers and their relations with smoking-related variables. 37 heavy smokers (29 males, mean age 47.19 ± 7.22 years) and 37 non-smoking healthy controls (29 males, mean age 46.95 ± 8.45 years) participated in the study. Subregional insular cortical thickness was evaluated and compared between the two groups. Correlation analysis was performed to investigate relationships between the insular cortical thickness and clinical characteristics in heavy smokers. There was no statistical difference on the cortical thickness in the left insula (*p* = 0.536) between the two groups while heavy smokers had a slightly thinner cortical thickness in the right insula (*p* = 0.048). In addition, heavy smokers showed a greater cortical thinning in the anterior (*p* = 0.0084) and superior (*p* = 0.0054) segment of the circular sulcus of the right insula as well as the inferior (*p* = 0.012) segment of the circular sulcus of the left insula. Moreover, the cortical thickness of the superior segment of the circular sulcus of the left insula was correlated negatively with nicotine severity (*r* = −0.423; *p* = 0.009) and the longer cigarette exposure was associated with the cortical thinning in the long insular gyrus and central sulcus of the right insula (*r* = −0.475; *p* = 0.003). Our findings indicate that chronic cigarette use is associated with region-specific insular thinning, which has the potential to improve our understanding of the specific roles of insular subregions in nicotine addiction.

## Introduction

Converging lines of evidence indicates that the insula is one of the critical brain regions implicated in the maintenance of smoking addiction (Naqvi and Bechara, 2010). Lesion studies demonstrated that damage to the insula in smokers led to a profound disruption of tobacco dependence, which was characterized by the ability to quit smoking easily and to remain a low urge to smoke (Naqvi et al., 2007; Abdolahi et al., 2017). Cue-reactivity related studies using functional magnetic resonance imaging (fMRI) showed insular activation and its correlation with the self-reported cigarette craving when smokers were exposed to smoking-related or abstinence-related cues (Warbrick et al., 2011; Engelmann et al., 2012; Zanchi et al., 2015; Dias et al., 2016; Janes et al., 2017). Resting-state fMRI (rs-fMRI) researches reported abstinence-induced functional connectivity changes in the insula and its association with the intensity of withdrawal-induced craving to cigarettes in smokers (Sutherland et al., 2013a,b; Huang et al., 2014; Maria et al., 2015; Bi et al., 2017b; Zhou et al., 2017; Faulkner et al., 2018). Progress has been made in understanding how the insular function influences smoking behavior, however, less is known about the association between the structural integrity of the insula and chronic cigarette use.

Structural MRI using voxel-based morphometry has been performed to examine the integrity of gray matter (GM) in smokers. The majority of studies revealed that smokers had smaller GM volume or lower GM density than non-smokers in various brain regions including the insula (Fritz et al., 2014; Wang et al., 2014; Hanlon et al., 2016; Stoeckel et al., 2016). One study, however, reported that smokers had greater GM density than non-smokers in the insula (Zhang et al., 2011). Cortical thickness, a topographical measurement indicating the integrity of cortical cytoarchitecture (Rakic, 1988), is considered to be more sensitive to evaluate GM structural changes than cortical volume (Hutton et al., 2009). Several studies demonstrated that smokers had lower cortical thickness in the frontal, temporal and parietal cortices, as well as the insula (Kuhn et al., 2010; Karama et al., 2015; Li et al., 2015; Durazzo et al., 2018). However, no differences in the insular cortical thickness have also been reported in smokers (Kuhn et al., 2010; Morales et al., 2014). The heterogeneous findings in the insular volume/density/cortical thickness may be attributed to variabilities in clinical and demographic variables (e.g., varying age ranges, smoking histories, and gender ratios) and different analysis methods. Inconsistencies may also be due to the structural and functional heterogeneity of the insula. Based on its cyto-and chemo-architectonic features as well as its anatomical and functional connectivity patterns, the insula can be parcellated into several subregions (Mesulam and Mufson, 1982; Afif and Mertens, 2010; Deen et al., 2011; Kelly et al., 2012). These insular subregions have distinct connections and functions and may have unique roles in maintaining addiction (Naqvi and Bechara, 2010; Droutman et al., 2015). Generally, the posterior insula connecting with the primary and secondary somatosensory regions, is associated with integrating somatosensory, vestibular and motor information, whereas the anterior insula connecting with the prefrontal cortex, anterior cingulate cortex and limbic regions, is involved in integrating autonomic and visceral information into emotional and motivational functions (Kurth et al., 2010b; Chang et al., 2013).

Based on previous findings of effects of cigarette smoking on the insular integrity, we hypothesized that heavy smokers would have a thinner cortical thickness in the insula than non-smokers. Given that the structural and functional heterogeneity of the insula, we further hypothesized that chronic cigarette smoking may have a differential effect on the cortical thickness of insular subregions. Therefore, in this study, to test the hypotheses, we evaluated the subregional insular cortical thickness according to the Destrieux Atlas (Destrieux et al., 2010) provided in Freesurfer and assessed its associations with clinical characteristics in heavy smokers.

## Materials and Methods

### Subjects

Seventy four right-handedness subjects, including 37 heavy cigarette smokers (29 males, aged 35–58 years) and 37 non-smoking healthy controls (29 males, aged 32–58 years), were enrolled in this study. The Mini International Neuropsychiatric Interview was used to screen for psychiatric and non-psychiatric medical disorders (Sheehan et al., 1998). All subjects had no history of neurological or psychiatric diseases, drug abuse or dependence (other than nicotine dependence for heavy smokers), or mental retardation. None of them self-reported daily alcohol consumption and these two group subjects had similar body mass index. Heavy smokers met the DSM-IV criteria for nicotine dependence and smoked at least 20 cigarettes per day for at least the past 5 years and had no period of smoking abstinence longer than 3 months in the past years. The Fagerström Test for Nicotine Dependence (FTND) was used to evaluate the severity of nicotine addiction (Heatherton et al., 1991). When heavy smokers came into the study, no special instruction was given regarding whether they should smoke or not. The brief questionnaire of smoking urges was used to confirm that none of the heavy smokers felt smoking urge or experience any withdrawal symptoms during their MRI scans. We refer such state of the heavy smokers when they were in the scanner as spontaneous, rather than abstinence or satiety. The non-smoking healthy controls had smoked less than 5 cigarettes in their lifetime. Table 1 lists the detailed information for subjects in each group.

**TABLE 1:** Demographic information for subjects in each group and between-group comparisons.

	**HS (*n* = 37)**	**NS (*n* = 37)**	*p* value
	**(Mean ± SD)**	**(Mean ± SD)**	
Age (years)	47.19 ± 7.22	46.95 ± 8.45	0.89
Gender (male/female)	29/8	29/8	1.00
Years of education (years)	8.46 ± 2.23	9.05 ± 3.04	0.34
Age at first smoking (years)	21.16 ± 5.39	–	–
Duration of smoking (years)	26.03 ± 8.93	–	–
Cigarettes per day	37.16 ± 10.11	–	–
Pack years	48.84 ± 20.63	–	–
FTND scores	8.89 ± 0.70	–	–

*Two-sample two tailed *t-* test was used for age and years of education comparisons between HS and NS. Values are expressed as mean ± standard deviation (SD). Pack years: duration of smoking × cigarettes smoked per day/20. HS, heavy smokers; NS, non-smokers; FTND, Fagerström test for nicotine dependence.*

This study adhered to the Declaration of Helsinki, and all procedures were approved by the Medical Ethics Review Board of Zhongnan Hospital, Wuhan University. Written informed consents were obtained from all participants before MRI scanning.

### Image Acquisition

All subjects underwent a high-resolution 3D T1-weighted structural MRI using a 3.0T Siemens scanner (Tim-Trio, Erlangen, Germany) with a standard birdcage head coil. A foam pad was used to minimize head movement and scanner noises. Structural MR images were obtained using a MPRAGE pulse sequence with the following parameters: repetition time=1900 ms; echo time=2.1 ms; inversion time=900 ms; flip angle=9°; field of view=256 × 256 mm^2^; data matrix=256 × 256; slices=160; 1 mm slice thickness without gap and voxel size=1 × 1 × 1 mm^3^. All 3D T1-weighted MPRAGE images were visually evaluated by two neuroradiologists to exclude pathological findings and none was excluded.

### Cortical Thickness Analysis

The 3D T1-weighted MPRAGE images were processed by Freesurfer (version 6.0.0)^1^. Procedures for cortical reconstruction have been described elsewhere (Dale et al., 1999; Fischl and Dale, 2000). In brief, the signal inhomogeneity of images was corrected and non-brain tissue was removed. White matter was first segmented to establish the gray-white matter interface. A tessellation of the gray-white matter surface was formed and the tessellation was then grown outward toward the intensity gradient which separates GM from cerebrospinal fluid to construct the pial surface. To check the accuracy in segmentation, the gray-white matter and pial surfaces for each subject were visually inspected in 2D coronal and axial slices overlaid on the T1-weighted images. Cases were deemed inaccurate if the inaccurate tissue delineation persisted for larger than 6 consecutive coronal or axial slices and the gray-white matter or pial surfaces were manually corrected and the cortical surfaces were reconstructed again (Iscan et al., 2015; Perlman et al., 2017). Finally, the cortical surfaces were transformed to a spherical coordinate system to align sulcal and gyral features across subjects. For each point on the gray-white matter surface, the shortest distance to the pial surface was calculated. In the same way, the shortest distance from every point on the pial surface to the gray-white matter surface was also measured. Cortical thickness was assessed by averaging these two distances.

**FIGURE 1:**
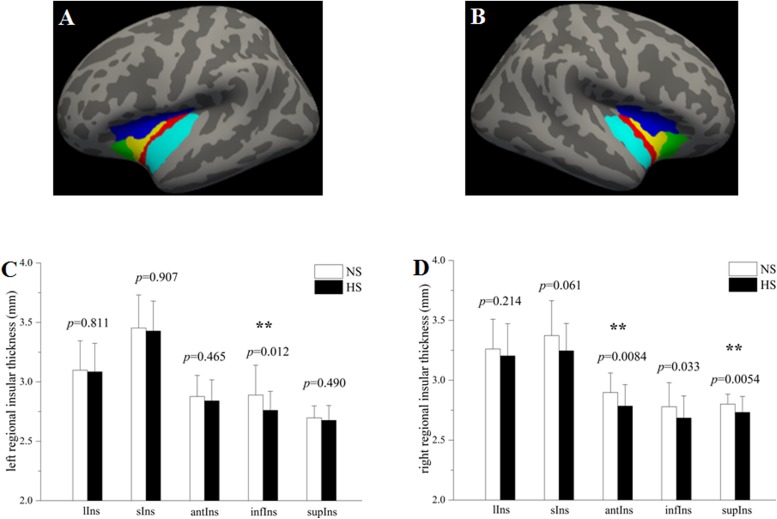
Group comparisons in the subregional insular cortical thickness between heavy smokers (HS) and non-smokers (NS). The left **(A)** and right insula **(B)** were parcellated into five subregions, respectively. Compared with non-smokers, heavy smokers had a significantly lower cortical thickness in the left infIns (*p* = 0.0083) while no statistical differences on other left insular subregions **(C)**, as well as a cortical thinning in the right antIns (*p* = 0.0057) and supIns (*p* = 0.0036) while no differences on other right insular subregions **(D)**. ^∗∗^*p <* 0.05 after false discovery rate corrected. lIns: the long insular gyrus and central sulcus of the insula (red); sIns: short insular gyri (yellow); antIns: anterior segment of the circular sulcus of the insula (green); infIns: inferior segment of the circular sulcus of the insula (cyan); supIns: superior segment of the circular sulcus of the insula (blue). The segmentation scheme for the insula was based on the Destrieux atlas provided in FreeSurfer (http://surfer.nmr.mgh.harvard.edu/fswiki/CorticalParcellation).

To evaluate subregional insular cortical thickness, the insula was parcellated into five subregions according to the Destrieux atlas (Destrieux et al., 2010) provided in Freesurfer^2^. In this atlas, the insula is limited by the circular sulcus and divided into superior, anterior and inferior segments. The central sulcus of the insula is divided into the short insular gyri and the long insular gyrus. The spatial distribution of insular subregions is displayed in Figures 1A,B. The whole insular cortical thickness was determined by averaging the subregional insular cortical thickness weighted by the respective surface areas of those subregions.

### Statistical Analysis

Statistical analysis was conducted with SPSS 20.0 (SPSS Statistics, IBM). Two-sample two-tailed *t* test was performed to detect differences in age and years of education between the two groups. Cortical thickness extracted from five insular subregions and intracranial volume (ICV) were imported into SPSS 20.0. Analysis of covariance (ANCOVA) controlling for the possible effects of age, gender, years of education, and ICV was carried out to evaluate between-group differences on the cortical thickness. For ICV, only age, gender, and years of education were covaried. To protect against false positive findings, a false discovery rate (FDR) multiple comparisons correction (Benjamini and Hochberg, 1995) with significant level at *q* = 0.05 was employed in the study. Cohen’s *d* was also calculated to measure effect sizes for pair-wised comparisons. In heavy smokers, Pearson correlation analysis was used to detect correlations between the cortical thickness and smoking-related variables (i.e., FTND, cigarettes smoked per day and pack years). Given the fact that effects of age, age at first smoking and duration of smoking on the cortical thickness may be confound each other, two-tailed partial correlation analysis controlling for age at first smoking was performed to reveal correlations between the cortical thickness and duration of smoking. A *p <* 0.05 (uncorrected) was considered statistically significant.

## Results

### Demographic Characteristics

Table 1 lists the demographic information for heavy smokers and non-smokers. There were no statistical differences on age (*p* = 0.89), gender (*p* = 1.00) or years of education (*p* = 0.34) between the two groups. On average, heavy smokers started smoking at 21.16 years (range: 14–34 years). They smoked 37.16 cigarettes per day (range: 20–60 cigarettes) and the average FTND score was 8.87 (range: 8–10), indicating heavy nicotine dependence. The heavy smokers had smoked for 26.03 years (range: 10–40 years).

### Whole Insular Cortical Thickness Analysis

**FIGURE 2:**
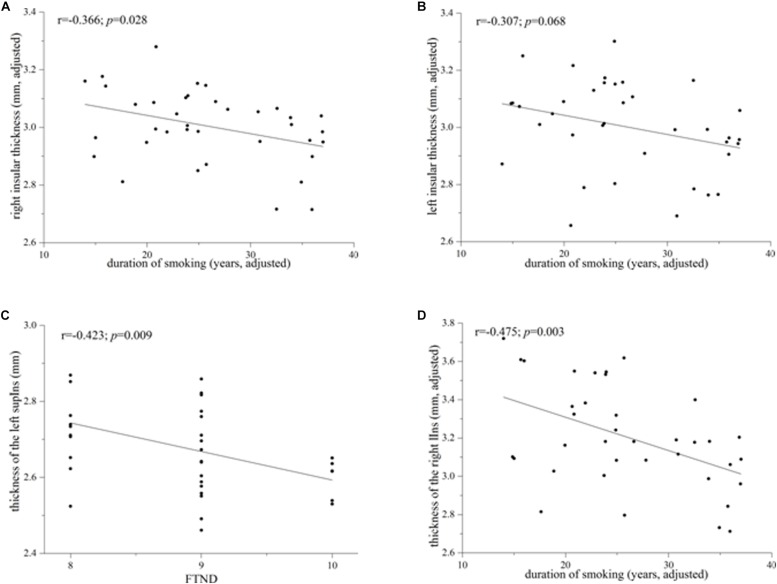
Correlations between the whole/subregional insular cortical thickness and smoking-related variables in heavy smokers. **(A)** The whole right insular cortical thickness was associated negatively with duration of smoking after correction for the age at first smoking (*r* = –0.366; *p* = 0.028). **(B)** The left whole insular cortical thickness had a trend toward negative correlation with duration of smoking when controlling for the age at first smoking (*r* = –0.307; *p* = 0.068). **(C)** Cortical thickness of the left superior segment of the circular sulcus of the insula (supIns) was correlated negatively with Fagerström Test for Nicotine Dependence (*r* = –0.423; *p* = 0.009). **(D)** Cortical thickness of the right long insular gyrus and central sulcus of the insula (lIns) was associated negatively with duration of smoking after correction for the age at first smoking (*r* = –0.475; *p* = 0.003).

Heavy smokers and non-smokers were equivalent on the ICV (heavy smokers: 1622.68 ± 179.74 ml; non-smokers: 1651.36 ± 191.24 ml; *p* = 0.429; Cohen’s *d* = 0.155). There was no significant difference on the left insular cortical thickness between the two groups (heavy smokers: 3.002 ± 0.158 mm; non-smokers: 3.029 ± 0.175 mm; *p* = 0.536; Cohen’s *d* = 0.162). However, heavy smokers had a slightly lower cortical thickness in the right insula than non-smokers (heavy smokers: 3.003 ± 0.131 mm; non-smokers: 3.080 ± 0.177 mm; *p* = 0.048; Cohen’s *d* = 0.495). In heavy smokers, after correction for the age at first smoking, the right insular cortical thickness had a negative association with duration of smoking (*r* = −0.366, *p* = 0.028; Figure 2A) while the left insular cortical thickness had a trend toward negative correlation with duration of smoking (*r* = −0.307, *p* = 0.068; Figure 2B).

### Subregional Insular Cortical Thickness Analysis

Although there was no significant difference on the cortical thickness in the left insula between the two groups, subregional insular cortical thickness analysis revealed that heavy smokers had a significant cortical thinning in the inferior segment of the circular sulcus of the left insula (*p* = 0.012 survived FDR correction at *q* < 0.05; Figure 1C). No differences on the cortical thickness were detected in other left insular subregions between the two groups (*p* > 0.05; Figure 1C). As for the right insular subregions, heavy smokers showed a significantly lower cortical thickness in the anterior (*p* = 0.0084) and superior (*p* = 0.0054) segment of the circular sulcus of the insula, surviving FDR correction at *q* < 0.05 (Figure 1D). Table 2 demonstrates the detailed information about subregional insular cortical thickness for heavy smokers and non-smokers and their comparisons. Correlation analysis revealed that the cortical thickness of the superior segment of the circular sulcus of the left insula was correlated negatively with FTND (*r* = −0.423, *p* = 0.009; Figure 2C) in heavy smokers. We also found the cortical thickness of the long insular gyrus and central sulcus of the right insula was associated negatively with duration of smoking (*r* = −0.475, *p* = 0.003; Figure 2D) after controlling for the age at first smoking in heavy smokers.

**TABLE 2:** Subregional insular thickness for subject groups and their comparisons.

Regional insula	HS	NS	*F*-value	**Effect size**	*P*-value
	(*n* = 37)	(*n* = 37)		**(Cohen’s *d*)**	
**Left**					
lIns	3.085 ± 0.240	3.098 ± 0.247	0.057	0.053	0.811
sIns	3.428 ± 0.252	3.453 ± 0.278	0.014	0.094	0.907
antIns	2.840 ± 0.176	2.877 ± 0.177	0.540	0.210	0.465
infIns	2.760 ± 0.159	2.889 ± 0.250	6.612	0.616	**0.012**
supIns	2.676 ± 0.124	2.697 ± 0.100	0.481	0.186	0.490
**Right**					
lIns	3.203 ± 0.269	3.261 ± 0.248	1.570	0.224	0.214
sIns	3.246 ± 0.229	3.373 ± 0.291	3.626	0.485	0.061
antIns	2.785 ± 0.179	2.897 ± 0.163	7.367	0.654	**0.0084**
infIns	2.685 ± 0.184	2.779 ± 0.201	4.751	0.488	0.033
supIns	2.732 ± 0.133	2.801 ± 0.082	8.253	0.624	**0.0054**

*Analysis of covariance (ANCOVA) with age, gender, years of education and intracranial volume as covariates was used to detect between-group differences on the subregional insular cortical thickness. Bold typeface indicates significant between-group differences after FDR corrected at *q* < 0.05. Values shown for each group are mean and standard deviation. HS, heavy smokers; NS, non-smokers; lIns, long insular gyrus and central sulcus of the insula; sIns, short insular gyri; antIns, anterior segment of the circular sulcus of the insula; infIns, inferior segment of the circular sulcus of the insula; supIns, superior segment of the circular sulcus of the insula.*

## Discussion

In this study, we particularly focused on the insula as its vital role in maintaining the addiction to cigarette smoking. Although the insular volume/density/cortical thickness have been studied extensively in smokers, inconsistent results about the insular integrity were reported, which may be attributed to the anatomical and functional heterogeneity of the insula. Therefore, it is of utmost importance to study the region-specific smoking effects on the insula. Our investigation of heavy smokers revealed that there was no significant difference on the left insular cortical thickness while a slight cortical thinning in the right insula. However, when the insula was subdivided into five subregions according to the Destrieux Atlas, heavy smokers showed a significantly lower cortical thickness in the anterior and superior segment of the circular sulcus of the right insula, and the inferior segment of the circular sulcus of the left insula. Our data suggest that chronic cigarette smoking is associated with the different effects on insular subregions, which may help us to understand the differential roles of insular subregions in cigarette addiction.

Although several studies have demonstrated alterations in the insular cortical thickness in smokers, the results were mixed. For example, cortical thinning (Li et al., 2015; Durazzo et al., 2018) and no cortical differences (Kuhn et al., 2010; Morales et al., 2014) in the insula were reported in smokers when compared to non-smokers. The reasons for the heterogeneity in these findings are unclear but may be partly due to the structural and functional heterogeneity of the insula and differences in clinical characteristics of the samples. Another non-negligible reason is the different analysis methods. Li et al. (2015) and Kuhn et al. (2010) used the surface-based morphometry (SBM) method, which analyzed whole-brain cortical thickness data unconstrained by *a priori* structure. Durazzo et al. (2018) and Morales et al. (2014) conducted the region-wised analyses with *a priori* regions-of-interest according to the Desikan-Killiany (DK) atlas (Desikan et al., 2006). Although SBM is a data-driven method without *a priori* hypothesis, it may miss some subtle alterations of cortical thickness for multiple comparisons correction. Compared to the DK atlas, the Destrieux atlas used in our study offers more and smaller insular subregions, which has been shown strong test–retest reliability (Iscan et al., 2015). Therefore, subtle region-specific changes in the insular cortical thickness can be detected using a higher spatial resolution of the insular atlas. To our knowledge, this is the first study to explore region-specific effects of chronic cigarette smoking on the insular cortical thickness in heavy smokers.

The finding of a lower cortical thickness of the right insula in heavy smokers is in line with previous studies in smokers. For example, when compared to non-smokers, decreased GM volume/density (Fritz et al., 2014; Wang et al., 2014; Hanlon et al., 2016; Stoeckel et al., 2016) and cortical thinning (Li et al., 2015) in the insula were found in smokers. In fact, reduced insular cortical thickness is a common finding in other substance use disorders. A lower cortical thickness of the insula has been reported in cocaine (Makris et al., 2008), marijuana (Lopez-Larson et al., 2011) and polysubstance abusers (Tanabe et al., 2013) as well as in alcoholists (Durazzo et al., 2013) and heroin-dependent subjects (Li et al., 2014). Interestingly, in our study, the cortical thinning was only found in the right insula, indicating that the right insula may be more relevant to smoking behavior than the left insula. Insular structural and functional alterations were often detected in the right hemisphere in smokers. The right insular cortical thickness was found to be negatively associated with cigarette exposure and craving in young adult smokers (Morales et al., 2014). Lesions to the right or left insula can disrupt the addiction behaviors in smokers, however, the possibility of having a disruption of smoking addiction caused by a lesion in the right insula may be higher than that in the left insula (Naqvi et al., 2007). Resting-state functional connectivity between the right anterior insula and ventromedial prefrontal cortex was associated with tobacco craving and alexithymia in adult smokers (Sutherland et al., 2013b). Of course, the left insula was also involved in cigarette dependence (Stoeckel et al., 2016). Taken together, we speculate that cigarette smoking like other substance use disorder is also related to the cortical thinning of the insula and future studies are needed to determine the different roles of the right and left insula in smoking behavior.

There was no statistical difference on the cortical thickness in the left insula and a slight cortical thinning in the right insula, however, we cannot rule out possible differences in subregional insular cortical thickness between heavy smokers and non-smokers for the structural and functional heterogeneity of the insula. Therefore, we explored region-based patterns for cortical thinning of the insula using the Destrieux atlas. Region-specific insular cortical thickness analysis revealed that heavy smokers had a significantly lower cortical thickness in the anterior and superior segment of the circular sulcus of the right insula as well as the inferior segment of the circular sulcus of the left insula, suggesting that chronic cigarette smoking has regional effect on the insular integrity. Anatomically, the anterior and superior segment of the circular sulcus of the insula, locating in front of the central insular sulcus, is considered as the ventral and dorsal anterior insular cortex, respectively (Shelley and Trimble, 2004; Gogolla, 2017). The ventral anterior insula has reciprocal connections to primarily limbic regions, and is functionally related to salience detection and interoceptive awareness (Seeley et al., 2007). The dorsal anterior insula is found to be connected to the dorsal anterior cingulate cortex and prefrontal cortex, and is involved in high-level cognitive control and attentional processes (Dosenbach et al., 2007). Thus, the anterior insula is considered to be associated with reward, affective and cognitive processes. Lower GM density in adult smokers was found in the anterior insula extending into the inferior frontal and temporal cortices (Stoeckel et al., 2016). Although there was no difference on the cortical thickness in the right anterior insula in young adult smokers, the severity of the urge to smoking and cigarette dependence (pack years) were associated negatively with the cortical thickness of the right ventral anterior insula (Morales et al., 2014). Moreover, resting-state fMRI studies revealed that smokers had reduced resting-state functional connectivity between the anterior insula and its interconnected brain regions commonly involved in craving and cognitive control (Sutherland et al., 2013b; Bi et al., 2017a). Thus, structural and functional alterations of the anterior insula were implicated in smoking addiction, including the pathological incentive salience, tracking withdrawal-related bodily states and dys-executive control of addictive behavior.

The inferior segment of the circular sulcus of the insula is a part of posterior insula that has strong connections with primary and secondary somatosensory cortices as well as the supplementary motor area (Kurth et al., 2010a). Abnormalities of the posterior insula may trigger overinterpretations of pain sensations and then facilitate relapse (Droutman et al., 2015), which would be consistent with a claim that an increased brain sensitivity to stressors contributes to substance relapse (Sinha, 2001). Thus, within this framework, the reduced cortical thickness in the posterior insula may express a neurobiological vulnerability predisposing people to the initiation of addictive behaviors and a structural factor contributing to the perpetuation of addictive behaviors (Gardini and Venneri, 2012). In fact, smaller GM volume in the posterior insula was also revealed in cocaine and heroin abusers (Gardini and Venneri, 2012). Thus, we assume that reduction in the cortical thickness of the posterior insula in heavy smokers may underlie a dysfunction of interoceptive and somatic processes as well as an alterations of visceral and homeostatic processes, which may enhance the urge to take drugs, alter emotional response and facilitate loss of control.

Importantly, in heavy smokers, we demonstrated the cortical thickness of the right insula (particularly the long insular gyrus and central sulcus of the right insula) was correlated negatively with duration of smoking, indicating the longer nicotine exposure, the lower cortical thickness in the right insula. In addition, the negative correlation between the cortical thickness of the superior segment of the circular sulcus of the left insula and FTND may indicate that the more nicotine severity, the thinner of the insula in heavy smokers. Negative associations between the cortical thickness and cigarette exposure/nicotine severity may be due to the neurotoxic effects of nicotine or other constituents of tobacco smoking. GM volume/density in the insula was lower in adult smokers (Makris et al., 2008; Fritz et al., 2014; Li et al., 2015; Hanlon et al., 2016) and correlated negatively with duration of smoking (Stoeckel et al., 2016). Although no differences in insular thickness were reported in young smokers, cigarette exposure was associated negatively with the cortical thickness of the right insula (Morales et al., 2014). Cigarette exposure or nicotine severity was also revealed to associated with the reduced GM volume/density in brain regions such as the medial prefrontal cortex (Hanlon et al., 2016), anterior cingulate cortex (Bu et al., 2016) and with the decreased white matter integrity in the prefrontal white matter (Zhang et al., 2011) and anterior corpus callosum (Lin et al., 2013). Thus, the findings of our study are consistent with previous studies and provide further evidence of the effects of chronic cigarette exposure on the cortical thinning in the insula in adult smokers.

This study has several limitations. First, the cross-sectional design of this study only demonstrates associations not causal relationships between the insular cortical thickness and nicotine dependence. We cannot be certain about whether the lower insular cortical thickness was a consequence of chronic cigarette exposure or a cause of nicotine dependence. We also cannot exclude the possibility that the lower insular cortical thickness may have existed before heavy smokers begun smoking and potentially be a marker of vulnerability to nicotine dependence. Second, possible sex-specific differences in response to chronic cigarette exposure may exist. Although we matched the sex proportion between the two groups, we did not assess the sex-specific differences on the insular cortical thickness because of the high male-to-female ratios and relatively small number of female subjects. Further studies with large female smokers should be made to investigate the sex-specific effects of chronic cigarette smoking on subregional insular cortical thickness. Third, we cannot rule out the possible influences from alcohol as alcohol consumption was not quantitatively evaluated. Although most subjects self-reported no daily alcohol consumption, the possibility of false reporting or deceptive reporting could not be excluded. Owing to high rate of drinking in smokers, it is reasonable to assume that smokers may drink at higher levels than non-smokers. The interaction effects of alcohol use and cigarette smoking on the insular cortical thickness should be investigated in future studies. Finally, although epidemiological studies showed that chronic cigarette smokers have poorer neurocognition compared with non-smokers (Durazzo et al., 2012), we cannot associate neuroimaging findings with cognition performances due to the lack of the cognition battery. Future MRI studies with neurocognition will help us to understand this condition.

In summary, we found the region-specific changes of insular cortical thickness and its associations with cigarette exposure and nicotine severity in heavy smokers. These findings shed new insights into the neurobiology of nicotine addiction and have the potential to improve our understanding of the pathogenesis of region-specific changes of the insula in heavy smokers. Future studies are required to investigate relationships between insular subregional structural and functional networks changes induced by chronic cigarette smoking.

## Data Availability

The datasets generated for this study are available on request to the corresponding author.

## Ethics Statement

This study was carried out in accordance with the recommendations of “name of guidelines, name of committee” with written informed consent from all subjects. All subjects gave written informed consent in accordance with the Declaration of Helsinki. The protocol was approved by the “Medical Ethics Review Board of Zhongnan Hospital, Wuhan University.”

## Author Contributions

FL, GW, and HL conceived and designed the study. GW and LZ contributed to the acquisition of data. FL undertook the MRI data analysis and drafted the manuscript. All authors critically reviewed the content.

## Conflict of Interest Statement

The authors declare that the research was conducted in the absence of any commercial or financial relationships that could be construed as a potential conflict of interest.

## Footnotes

1 http://surfer.nmr.mgh.harvard.edu

2 http://surfer.nmr.mgh.harvard.edu/fswiki/CorticalParcellation
